# Vitamin B12 deficiency and altered one-carbon metabolites in early pregnancy is associated with maternal obesity and dyslipidaemia

**DOI:** 10.1038/s41598-020-68344-0

**Published:** 2020-07-06

**Authors:** Antonysunil Adaikalakoteswari, Catherine Wood, Theresia H. Mina, Craig Webster, Ilona Goljan, Yonas Weldeselassie, Rebecca M. Reynolds, Ponnusamy Saravanan

**Affiliations:** 1 Department of Biosciences, School of Science and Technology, Nottingham Trent University, Clifton, Nottingham UK; 2 Warwick Medical School, Gibbet Hill, University of Warwick, Warwick, Coventry, UK; 3 Academic Department of Diabetes and Metabolism, George Eliot Hospital, Nuneaton, UK; 4 Centre for Cardiovascular Science, University of Edinburgh, Edinburgh, UK; 5 Heartlands Hospital, Birmingham, UK

**Keywords:** Metabolic disorders, Nutrition disorders

## Abstract

Vitamin B12 (B12) is a micronutrient essential for one-carbon (1C) metabolism. B12 deficiency disturbs the 1C cycle and alters DNA methylation which is vital for most metabolic processes. Studies show that B12 deficiency may be associated with obesity, insulin resistance and gestational diabetes; and with obesity in child-bearing women. We therefore hypothesised that the associations between B12 deficiency, BMI and the metabolic risk could be mediated through altered 1C metabolites in early pregnancy. We explored these associations in two different early pregnancy cohorts in the UK (cohort 1; n = 244 and cohort 2; n = 60) with anthropometric data at 10–12 weeks and plasma/serum sampling at 16–18 weeks. B12, folate, total homocysteine (tHcy), methionine, MMA, metabolites of 1C metabolism (SAM, SAH) and anthropometry were measured. B12 deficiency (< 150 pmol/l) in early pregnancy was 23% in cohort 1 and 18% in cohort 2. Regression analysis after adjusting for likely confounders showed that B12 was independently and negatively associated with BMI (Cohort 1: β = − 0.260, 95% CI (− 0.440, − 0.079), p = 0.005, Cohort 2: (β = − 0.220, 95% CI (− 0.424, − 0.016), p = 0.036) and positively with HDL cholesterol (HDL-C) (β = 0.442, 95% CI (0.011,0.873), p = 0.045). We found that methionine (β = − 0.656, 95% CI (− 0.900, − 0.412), p < 0.0001) and SAH (β = 0.371, 95% CI (0.071, 0.672), p = 0.017) were independently associated with triglycerides. Low B12 status and alteration in metabolites in 1C metabolism are common in UK women in early pregnancy and are independently associated with maternal obesity and dyslipidaemia. Therefore, we suggest B12 monitoring in women during peri-conceptional period and future studies on the pathophysiological relationship between changes in 1C metabolites and its association with maternal and fetal outcomes on larger cohorts. This in turn may offer potential to reduce the metabolic risk in pregnant women and their offspring.

## Introduction

The rise in obesity is a global challenge and is rapidly increasing in reproductive age. This influences obesity and increased risk of adverse pregnancy outcomes during pregnancy, which reinforces the risk to childhood obesity and other non-communicable diseases in adult life^1^. It is known that maternal macronutrients influence obesity, but emerging evidence suggest micronutrient deficiency such as vitamin B12, could play a role in adipogenesis^2,3^. B12 is involved in 1C metabolism and supplies the methyl groups required for the DNA methylation, which is key for maternal health and optimal fetal growth and development. Deficiency in B12 alters the levels of total homocysteine (tHcy), methionine, methylmalonic acid (MMA) and metabolites of 1C cycle including S-adenosyl methionine (SAM) and S-adenosyl homocysteine (SAH), which is associated with various adverse pregnancy outcomes and cardiovascular complications^4^. Since epigenetic modifications play a major role on the pathogenesis of many disease states, recent research predicts that metabolites of 1C metabolism might have an intermediate effect in adverse metabolic outcomes^5^.

Several observational and epidemiological studies have shown that low B12 is associated with high BMI^6^, insulin resistance (IR)^7^, increasing severity of glucose tolerance^8^ and adverse lipid profiles in type 2 diabetes (T2D)^9^. Supporting this, a recent Mendelian Randomization study^10^ reported a possible causal relationship between B12 and fasting glucose and β-cell function. A series of studies during pregnancy in both South Asian^11,12^ and Caucasian^6^ have shown association of low B12 with obesity, IR and higher risk of GDM. In children and adolescents, increase in BMI^13^ and IR^14^ have been associated with low B12 levels. Furthermore, studies in early pregnancy have focussed on association between lower maternal micronutrient status (B12, folate, iron) with pre-pregnancy overweight and obesity in mothers^15^^,^^16^^,^^17^, with lower micronutrients in low-birth weight infants^18^ and with higher BMI and heart rate in children at age 5–6^19^. Our in vitro studies have shown that B12 deficiency in adipocytes altered DNA methylation levels^2^ of the transcription factors of lipid regulation^2^ and the adipose derived miRNAs targeting IR^3^ thereby suggesting that B12 deficiency dysregulates epigenetic mechanisms.

Though several studies have found an association between B12 and metabolic risk, there are very few studies on 1C metabolites in pregnancy. Our pilot study showed association of 1C metabolites with low B12 and increased triglycerides^20^. A study in Brazil reported that lower maternal B12 concentration is associated with higher tHcy and lower SAM:SAH in newborns^21^. A Danish cross-sectional study in non-pregnant adults found that plasma 1C metabolites positively associated with higher risk of metabolic dysfunction^22^. In addition, a recent study of mother-infant dyads identified changes in methylation patterns in 993 CpG sites, which were related to altered 1C metabolite indices over pregnancy, thereby suggesting the impact of different 1C metabolites on regulation of DNA methylation at multiple sites^23^. This evidence supports that altered 1C metabolites, adversely affect epigenetic mechanisms and metabolic health of both mother and offspring. Since underlying nutritional status influences the predictors of DNA methylation, studying these 1C biomarkers at early pregnancy, the embryonic development period involving complex epigenetic remodelling will extend our understanding of association of 1C metabolites with metabolic risk.

Furthermore, understanding the early life contributors will be crucial for delineating the risk factors and their mechanisms leading to maternal obesity and metabolic risk. This may inform targeted implementation of obesity prevention in early pregnancy. We therefore hypothesise that the associations between B12 deficiency, BMI and the metabolic risk could be mediated through altered 1C metabolites in early pregnancy. Our current study investigated the prevalence of B12 deficiency and the associated changes in tHcy, methionine, MMA and metabolites of 1C metabolism (SAM and SAH) during early pregnancy. We also report their association with maternal obesity and dyslipidemia.

## Research design and methods

### Study population

#### Cohort 1

The study was conducted in George Eliot Hospital, Nuneaton, UK. Pregnant women (n = 244) who met the inclusion criteria of women with singleton pregnancy, free of chronic diseases or any other pregnancy complications and delivered full-term (37–42 weeks of gestation) healthy infant were included in this study. All pregnant women provided blood samples between 16 and 18 weeks of their pregnancy as part of their routine care. Data on age, weight, smoking, alcohol, parity and socioeconomic status were obtained from pregnancy records. The samples were stored at − 80 °C and later analysed for metabolites. All methods were carried out in accordance with the approved ethical guidelines. The study was approved by the NHS Research Ethics Committee (12/LO/0239) and all subjects gave written informed consent.

#### Cohort 2

This study was conducted in the Antenatal Metabolic Clinic, Simpson’s Centre for Reproductive Health, Royal Infirmary of Edinburgh. Pregnant women (n = 60) identified during their first community midwifery visit having a BMI ≥ 40 and lean controls with BMI ≤ 25 were invited to participate in a prospective cohort study from 2008 to 2012^24^. Information on age, parity, smoking status and social and demographic data were collected. The main exclusions were pre-existing diabetes and major anomalies. Women who developed GDM or did not undergo an oral glucose tolerance test at 24–28 weeks’ gestation or developed pre-eclampsia or delivered < 37 weeks’ gestation were excluded from analyses. Venous blood samples were collected at 16–18 weeks’ gestation after an overnight fast and samples were stored at − 80 °C for later determination of metabolites. All research methods were performed in accordance with relevant guidelines and regulations. Ethical approval was obtained from the Lothian NHS Research Ethics Committee, and all subjects gave informed written consent (REC reference number 08/S1101/39)^25^.

### Analytical determinations

Serum B12 and folate were determined by electro-chemiluminescent immunoassay using a Roche Cobas immunoassay analyzer (Roche Diagnostics, UK). We used 150–660 pmol/l for serum B12 and 6–37 nmol/l for serum folate as normal ranges, respectively. The inter-assay coefficient of variations for B12 and folate were 3.9% and 3.7%, respectively^26^. B12 deficiency was defined as B12 levels below 150 pmol/l, borderline B12 deficiency as 150–220 pmol/l and folate deficiency as below 6 nmol/l. Excess folate is defined as > 37 nmol/l (upper limit of detection by the assay). Measurement of tHcy, methionine, MMA and metabolites of 1C metabolism (SAM and SAH) were determined in plasma by stable isotopic dilution analysis liquid chromatography using an API 6,500 QTrap tandem mass spectrometer (Applied Biosystems, UK)^20^. Glucose concentrations were measured by a hexokinase method (Randox Laboratories, Co. Antrim, UK), total cholesterol, HDL cholesterol (HDL-C), (Olympus Diagnostics, Watford, UK) and triglycerides (Alpha Laboratories, Eastleigh, UK) were measured by colorimetric methods.

### Statistical analysis

Continuous data are reported as mean ± standard deviation (SD). Categorical data are reported in percentages. The distributions of the data such as B12, folate, tHcy, MMA, methionine, SAM, SAH, cholesterol, triglycerides and HDL-C were skewed; these data were log-transformed before analyses. Student’s t-test was used for comparison of groups. Pearson’s correlation was used to compare the variables. Variables that showed significant associations were included as independent variables in the multiple linear regression analyses and adjusted for confounders such as age, smoking, alcohol, parity, BMI, B12, folate, tHcy, MMA, methionine, SAM and SAH. To facilitate comparison, dependent and independent variables were converted into standard deviation scores (SDS). The data are presented as SDS change in dependent variable per SDS change in BMI, triglycerides and HDL-C. All tests were two-sided, and p-values of < 0.05 were considered to be statistically significant. All analyses were performed using SPSS Statistics version 21 (IBM Corp, NY, USA)^26^.

## Results

### Baseline characteristics

The clinical characteristics of pregnant women from two cohorts are shown in Table 1. All women were recruited in early pregnancy at 16–18 weeks. Their booking BMI were collected at 10–12 weeks of gestation. Significant proportion of women were obese and reported higher rates of smoking and alcohol intake during pregnancy (Table 1). We found the rates of B12 and borderline B12 deficiency were 23 and 36% in cohort 1 and 18 and 43% in cohort 2, respectively. However, we did not observe any folate deficiency in either of these cohorts (Table 1).

**Table 1:** Clinical characteristics of pregnant women at early pregnancy.

Variables	Cohort 1 (n = 244)	Cohort 2 (n = 60)
**Lifestyle/demographics**		
Age (years)	27.6 ± 5.7	33.2 ± 4.4
Socio-economic status (Deprivation Quintile)	3.10 ± 1.27	2.52 ± 1.93
Smoking (%)	19.2	48.3
Alcohol (%)	16.4	76.5
Parity (%)		
0 1 > = 2	45.9 37.2 16.9	58.3 28.3 13.3
**Anthropometry**		
Booking visit BMI (kg/m^2^)	26.5 ± 5.5	32.6 ± 11.2
Obesity (%)	18.4	45.0
**Micronutrients**		
B12 (pmol/l)	223 ± 99	233 ± 100
B12 deficiency (%)	23.0	18.3
Folate (nmol/l)	25.8 ± 10.1	28.7 ± 11.6
Folate deficiency (%)	0	0
High folate levels (%)	3.3	6.6

Data are mean ± SD; Categorical values are presented as percentage.

### Association of B12 with metabolic risk factors in early pregnancy

**Figure 1:**
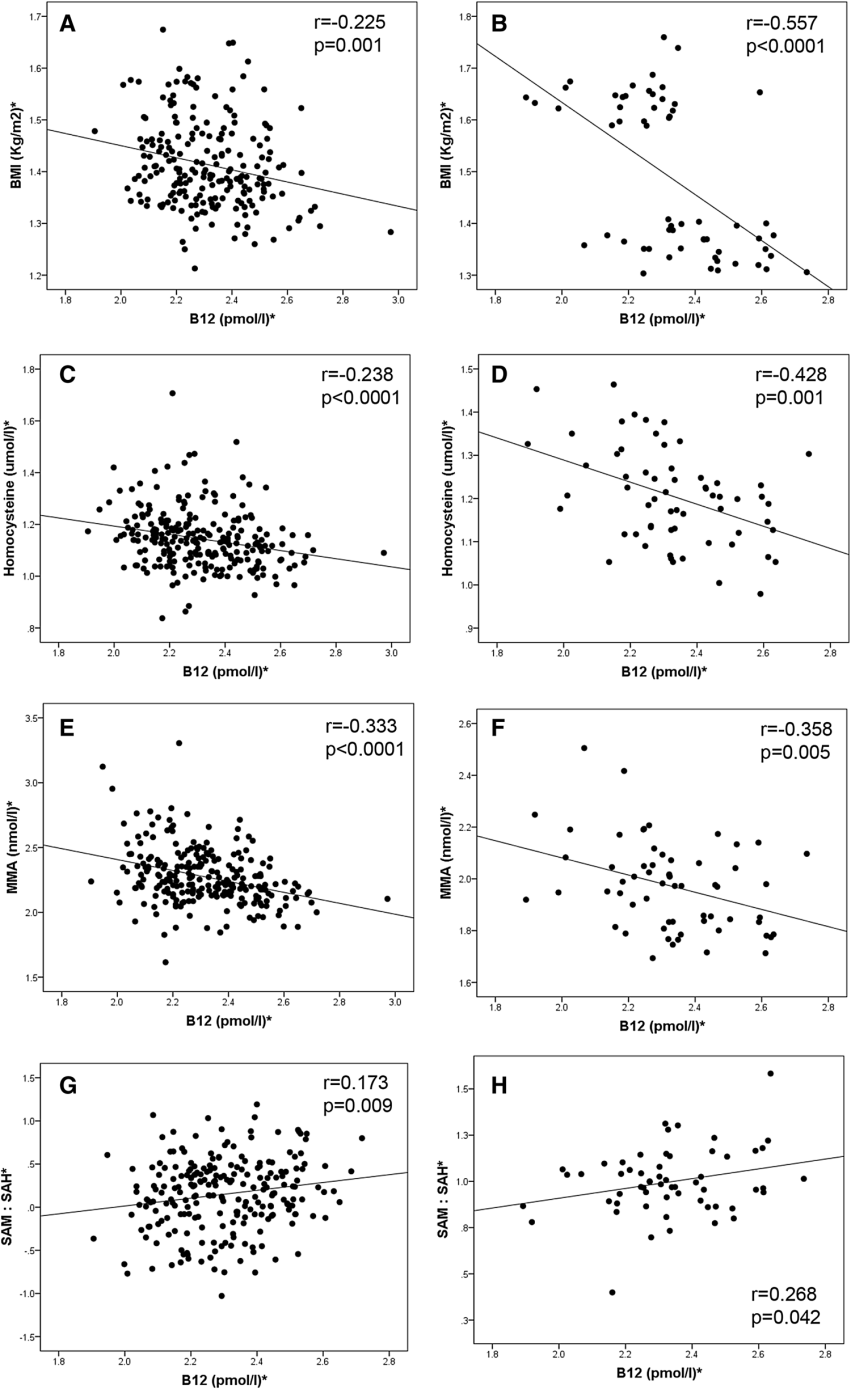
*Log transformed for statistical comparisons. Correlation between B12 and (**A**) BMI in cohort 1, (**B**) BMI in cohort 2, (**C**) Homocysteine in cohort 1, (**D**) Homocysteine in cohort 2, (**E**) MMA in cohort 1, (**F**) MMA in cohort 2, (**G**) SAM:SAH in cohort 1, (**H**) SAM:SAH in cohort 2. Cohort 1: n = 240; cohort 2: n = 60.

**Figure 2:**
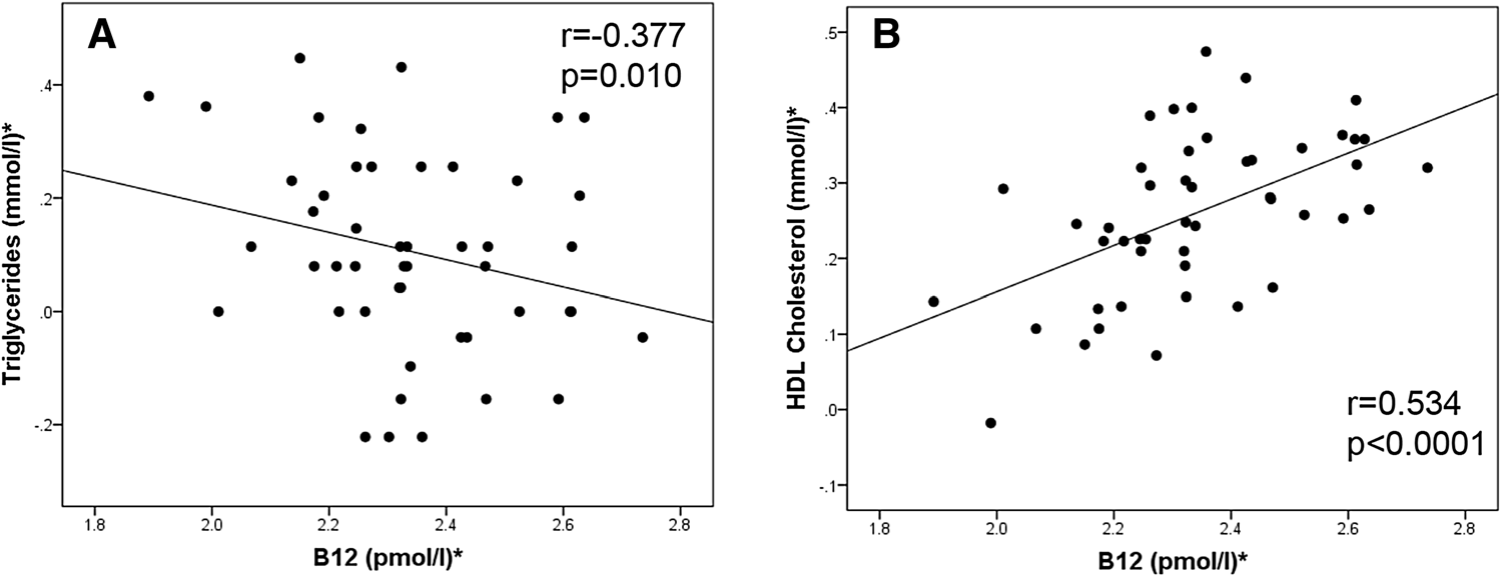
*Log transformed for statistical comparisons. Correlation between B12 and (**A**) Triglycerides in cohort 2, (**B**) HDL-C in cohort 2. Cohort 2: n = 60.

In both cohorts, B12 was negatively correlated with BMI, tHcy and MMA; and positively correlated with SAM/SAH (Fig. 1A–H). In addition, in cohort 2, there was evidence of association of B12 with lipid parameters such as negative correlation with triglycerides and positive correlation with HDL-C (Fig. 2A–B). To further examine the independent association of B12 and 1C metabolites with these risk factors, multiple regression analyses were carried out including the potential cofounders such as age, smoking, alcohol, parity, and socio-economic status. We found that B12 remained independently associated with BMI (Cohort 1: β = − 0.260, 95% CI (− 0.440, − 0.079), p = 0.005, Cohort 2: (β = − 0.220, 95% CI (− 0.424, − 0.016), p = 0.036) when adjusted for all co-variates (Table 2).

**Table 2:** Multiple regression analysis of B12 with BMI in early pregnancy.

BMI*	Cohort 1			Cohort 2		
	Β	95% CI	p	Β	95% CI	p
**Model 1**						
B12*	− 0.237	(− 0.377, − 0.097)	**0.001**	− 0.564	(− 0.787, − 0.314)	** < 0.0001**
**Model 2**						
Age	0.017	(− 0.011, 0.046)	0.229	− 0.033	(− 0.068, 0.001)	0.059
Smoking	− 0.191	(− 0.542, 0.161)	0.287	0.003	(− 0.266, 0.273)	0.980
Alcohol	0.264	(− 0.047, 0.575)	0.096	− 0.002	(− 0.030, 0.025)	0.855
Parity	0.138	(− 0.167, 0.444)	0.372	0.196	(− 0.043, 0.436)	0.0106
**Socio-economic status**	0.045	(− 0.066, 0.155)	0.426	0.404	(0.293, 0.515)	** < 0.0001**
B12*	− 0.187	(− 0.358, − 0.016)	**0.032**	− 0.185	(− 0.377, 0.007)	**0.059**
Folate*	− 0.002	(− 0.162, 0.158)	0.982	0.142	(− 0.090, 0.374)	0.222
**Model 3**						
Age	0.018	(− 0.010, 0.047)	0.205	− 0.020	(− 0.058, 0.018)	0.300
Smoking	− 0.201	(− 0.559, 0.156)	0.268	0.016	(− 0.299, 0.331)	0.918
Alcohol	0.210	(− 0.107, 0.526)	0.193	− 0.006	(− 0.034, 0.022)	0.683
Parity	0.145	(− 0.160, 0.451)	0.350	0.208	(− 0.036, 0.452)	0.092
**Socio-economic status**	0.069	(− 0.046, 0.183)	0.239	0.372	(0.245, 0.499)	** < 0.0001**
B12*	− 0.260	(− 0.440, − 0.079)	**0.005**	− 0.220	(− 0.424, − 0.016)	**0.036**
Folate*	− 0.008	(− 0.175, 0.158)	0.923	0.102	(− 0.156, 0.360)	0.427
Homocysteine*	− 0.054	(− 0.221, 0.112)	0.521	0.022	(− 0.223, 0.268)	0.853
MMA*	− 0.177	(− 0.363, 0.010)	0.064	− 0.171	(− 0.359, 0.016)	0.072
Methionine*	0.123	(− 0.093, 0.339)	0.263	0.100	(− 0.098, 0.299)	0.312
SAM*	0.145	(− 0.012, 0.303)	0.070	0.056	(− 0.110, 0.222)	0.500
SAH*	0.015	(− 0.175, 0.205)	0.877	0.072	(− 0.114, 0.258)	0.436

*Log transformed for statistical comparisons, β represents SDS change in the dependent variable per SDS change in the independent variable. SAM—S-adenosyl methionine, SAH—S-adenosyl homocysteine, MMA—Methyl malonic acid.

Model 1: Unadjusted.

Model 2: Model 1 + Adjusted for age, smoking, alcohol, parity, socio-economic status, B12, folate.

Model 3: Model 2 + Adjusted for homocysteine, MMA, methionine, SAM and SAH.

In addition, in cohort 2 (Table 3), in the fully adjusted model, B12 showed independent, positive associations with HDL-C (β = 0.442, 95% CI (0.011, 0.873), p = 0.045). We also found that low maternal B12 during early pregnancy was associated with higher triglycerides in the unadjusted model, but after adjustment for all potential confounders, this association was not significant. However, we found that methionine (β = − 0.656, 95% CI − 0.900, − 0.412, p < 0.0001) and SAH (β = 0.371, 95% CI: 0.071, 0.672, p = 0.017) were associated significantly with higher triglycerides after adjusting for the confounders. This implies that higher triglycerides in B12 deficient women during early pregnancy could be mediated by these metabolites.

**Table 3:** Multiple regression analysis of B12 with triglycerides and HDL-C at early pregnancy.

Cohort 2	Triglycerides*			HDL-C*		
	Β	95% CI	p	Β	95% CI	p
**Model 1**						
B12*	− 0.378	(− 0.660, − 0.095)	**0.010**	0.506	(0.268, 0.744)	** < 0.0001**
**Model 2**						
Age	− 0.061	(− 0.137, 0.015)	0.111	0.059	(− 0.005, 0.124)	0.069
Smoking	0.354	(− 0.153, 0.860)	0.165	0.128	(− 0.334, 0.589)	0.577
Alcohol	0.040	(− 0.011, 0.091)	0.122	− 0.013	(− 0.060, 0.035)	0.587
Parity	0.262	(− 0.267, 0.790)	0.321	− 0.082	(− 0.526, 0.362)	0.708
Socio-economic status	0.116	(− 0.243, 0.475)	0.515	− 0.104	(− 0.428, 0.220)	0.520
BMI*	− 0.058	(− 0.794, 0.678)	0.874	− 0.103	(− 0.668, 0.663)	0.970
B12*	− 0.317	(− 0.703, 0.068)	0.103	0.515	(0.161, 0.869)	**0.006**
Folate*	− 0.126	(− 0.591, 0.339)	0.584	− 0.210	(− 0.621, 0.200)	0.305
**Model 3**						
Age	− 0.056	(− 0.110, − 0.001)	0.046	0.065	(− 0.005, 0.135)	0.060
Smoking	0.520	(0.124, 0.917)	0.012	− 0.063	(− 0.614, 0.489)	0.817
Alcohol	0.034	(− 0.003, 0.070)	0.069	− 0.013	(− 0.064, 0.038)	0.599
Parity	0.162	(− 0.210, 0.534)	0.380	− 0.066	(− 0.534, 0.403)	0.776
Socio-economic status	0.033	(− 0.237, 0.303)	0.806	− 0.018	(− 0.379, 0.342)	0.917
BMI*	− 0.045	(− 0.638, 0.549)	0.878	− 0.123	(− 0.945, 0.699)	0.762
B12*	− 0.212	(− 0.529, 0.105)	0.182	0.442	(0.011, 0.873)	**0.045**
Folate*	− 0.284	(− 0.650, 0.082)	0.123	− 0.220	(− 0.702, 0.261)	0.357
Homocysteine*	0.240	(− 0.081, 0.561)	0.136	− 0.244	(− 0.681, 0.192)	0.262
MMA*	− 0.005	(− 0.267, 0.256)	0.966	− 0.085	(− 0.435, 0.264)	0.622
Methionine*	− 0.656	(− 0.900, − 0.412)	**< 0.0001**	0.113	(− 0.212, 0.437)	0.483
SAM*	0.252	(− 0.007, 0.511)	0.056	0.094	(− 0.262, 0.450)	0.592
SAH*	0.371	(0.071, 0.672)	**0.017**	0.043	(− 0.292, 0.378)	0.795

*Log transformed for statistical comparisons, β represents SDS change in the dependent variable per SDS change in the independent variable. SAM – S-adenosyl methionine, SAH—S-adenosyl homocysteine, MMA—Methyl malonic acid; Model 1: Unadjusted.

Model 2: Model 1 + Adjusted for age, smoking, alcohol, parity, socio-economic status, BMI, B12, folate.

Model 3: Model 2 + Adjusted for homocysteine, MMA, methionine, SAM and SAH.

## Discussion

Our study involving two cohorts with Caucasian women at early pregnancy from UK had three main findings. Firstly, vitamin B12 deficiency rates were 23% in cohort 1 and 18% in cohort 2. Secondly, low B12 level was associated with higher BMI and lower HDL-C. Thirdly, we found that lower levels of methionine and higher levels of SAH associated with higher triglycerides in these women at early pregnancy.

The prevalence of B12 deficiency in early pregnancy has been reported in very few studies. A recent systematic review showed that B12 deficiency were 21%, 19%, and 29% in the first, second and third trimesters, respectively^27^. We found similar rates of deficiency of B12 (cohort 1–23% & cohort 2–18%) in our cohorts in early pregnancy. The regression analysis in cohort 1, after correcting for multiple confounders, we found that B12 is independently associated with BMI whereas in cohort 2, in addition to B12, social deprivation was also associated with BMI. The association between BMI and B12 has been shown in other studies in pregnant women^15,16,17^ but also in adolescents^14^ and in women of childbearing age^28^. In addition, B12 deficiency may be causally related to dysglycaemia^9^. It is therefore conceivable that if women with higher prevalence of B12 deficiency enter pregnancy, it may predispose them to greater risk of obesity, dyslipidemia and hyperglycaemia. However, longitudinal studies are required to investigate whether low B12 in early pregnancy increases the risk of incident GDM.

In a recent meta-analysis, we reported that maternal low HDL-C and high triglycerides at different trimesters of pregnancy are associated with higher birthweight, higher risk of large for gestational age and macrosomia^29^. The present study showed that lower B12 was associated with lower HDL-C levels in early pregnancy. In our previous study of pregnant women at third trimester a similar association was seen^26^. Consistent to this, Knight et al.^6^ showed this relationship in women at 28 weeks of gestation in the UK and Saraswathy et al.^30^ in a non-pregnant adult population in India. In addition, our mechanistic studies in adipocytes indicate that in low B12 status, hypomethylation of the lipid transcription factors (SREBF1 and LDLR)^2^ and alteration of miRNAs^3^ could adversely regulate the adipogenic and lipogenic genes and promote dyslipidaemia.

We also observed that in addition to the effect of B12 deficiency on lipid regulation, metabolites such as lower methionine and higher SAH were associated with higher triglycerides. This was similar to our previous observation in a small study of 30 pregnant women in early pregnancy, where the tHcy and 1C metabolites (SAM, SAM:SAH) were independently associated with triglycerides^20^. A study in Netherlands in women of reproductive age, found that BMI was the strongest determinant of SAM and SAH concentrations^31^. Lind et al. showed in people with the metabolic syndrome, the 1C metabolites were determinants of markers of adiposity such as BMI, body fat percentage and IR^22^. Plasma SAH has been shown to be better indicator of cardiovascular disease than tHcy^32^ and in vitro models have shown that elevated SAH adversely altered adipocyte function and altered methylation^33^. In vascular disease, global DNA methylation was altered with a concomitant increase in plasma tHcy and SAH^34^. Of further interest, a recent study by Knight et al. demonstrated that five out of thirteen 1C metabolites changed over pregnancy and these metabolites were associated with altered methylation levels of genes involved in neuronal and developmental process in both maternal and cord blood^23^. These findings add to the evidence that alteration in the indices of 1C metabolism in B12 deficient status could change the methylation potential, mediate the epigenetic modifications and contribute to adverse lipid levels and metabolic outcomes. Thus, circulating levels of indices of 1C metabolism could be used as a surrogate for cellular level methylation capacity in early pregnancy. As the biomarkers of cellular methylation are related to health and disease and influenced by micronutrients, it would therefore be of interest to further study this hypothesis in human trials or animal studies to show whether B-vitamins supplementation in peri-conceptional period reduces metabolic risk of the offspring by altering these methylation biomarkers.

The strengths of this study are that we included two cohorts of pregnant women from UK at 16–18 weeks of gestation. Detailed information of lifestyle, socio-economic status and methylation biomarkers were available for both the cohorts, which allowed to control for multiple potential confounders. The rate of B12 deficiency and the factors associated with obesity in the two settings increases the generalizability of findings. However, we acknowledge the following limitations. This study is observational in a single time point, so causality cannot be established. The lack of associations observed in other indices of 1C metabolism may well be due to our sample size and hence future investigations should include larger sample size. We did not have lipid levels or holo-transcobalamin levels in the first cohort. However, the independent associations seen with B12 and BMI in both the cohorts as well as the associations with B12/methionine/SAH and HDL-C/triglyceride levels despite the smaller size of cohort 2, warrants further investigation in larger studies. Finally, our study focussed only on some of the indices of the methionine cycle (and serum folate). Future studies measuring the entire 1C metabolite pathway (including the trans-sulphuration pathway) in early pregnancy, may provide more insights into cellular methylation potential and other epigenetic mechanisms.

In summary, our novel study highlighted that underlying nutritional (B12) status is an effective modifier of some of the 1C metabolites in early pregnancy. B12 deficiency in early pregnancy is adversely associated with altered level of 1C metabolites, maternal obesity and dyslipidaemia. These findings may have vital implications for pregnant women and their offspring, especially if they are overweight and obese. Out study suggests that assessment of B12 levels for women planning pregnancy may be worthwhile. Future investigations focusing on metabolites involved in 1C cycle associated with maternal and fetal outcomes is warranted for targeted intervention and obesity prevention in both mother and offspring.

## Abbreviations

1CM: One-carbon metabolism
B12: Vitamin B12
GDM: Gestational diabetes
tHcy: Total homocysteine
IR: Insulin resistance
MMA: Methylmalonic acid
SAH: S-adenosyl homocysteine
SAM: S-adenosyl methionine
SD: Standard deviation
SDS: Standard deviation scores
T2D: Type 2 diabetes
